# Perception of gait patterns that deviate from normal and symmetric biped locomotion

**DOI:** 10.3389/fpsyg.2015.00199

**Published:** 2015-02-27

**Authors:** Ismet Handžić, Kyle B. Reed

**Affiliations:** REED Lab, Department of Mechanical Engineering, University of South FloridaTampa, FL, USA

**Keywords:** uncanny valley, gait perception, biped walking, passive dynamic walking, gait simulation, pathological gait, rehabilitation

## Abstract

This study examines the range of gait patterns that are perceived as healthy and human-like with the goal of understanding how much asymmetry is allowable in a gait pattern before other people start to notice a gait impairment. Specifically, this study explores if certain abnormal walking patterns can be dismissed as unimpaired or not uncanny. Altering gait biomechanics is generally done in the fields of prosthetics and rehabilitation, however the perception of gait is often neglected. Although a certain gait can be functional, it may not be considered as normal by observers. On the other hand, an abnormally perceived gait may be more practical or necessary in some situations, such as limping after an injury or stroke and when wearing a prosthesis. This research will help to find the balance between the form and function of gait. Gait patterns are synthetically created using a passive dynamic walker (PDW) model that allows gait patterns to be systematically changed without the confounding influence from human sensorimotor feedback during walking. This standardized method allows the perception of specific changes in gait to be studied. The PDW model was used to produce walking patterns that showed a degree of abnormality in gait cadence, knee height, step length, and swing time created by changing the foot roll-over-shape, knee damping, knee location, and leg masses. The gait patterns were shown to participants who rated them according to separate scales of impairment and uncanniness. The results indicate that some pathological and asymmetric gait patterns are perceived as unimpaired and normal. Step time and step length asymmetries less than 5%, small knee location differences, and gait cadence changes of 25% do not result in a change in perception. The results also show that the parameters of a pathologically or uncanny perceived gait can be beneficially altered by increasing other independent parameters, in some sense masking the initial pathology.

## 1. Introduction

In order to systematically generate a variety of altered gait dynamics to be rated for impairment and uncanniness by participants, we are using one measured healthy gait (for comparison) and sets of simulated gait models that are mathematically derived. Simulated gait models allow for consistency and precision of the altered gait parameters. This systematic change allows for a controlled experiment on the perception of specific gait changes. By using a passive dynamic walker (PDW) computational model, we are able to specifically examine changes in perception that arise from deviations in gait speed, knee location, spatial and temporal symmetry, foot roll-over shapes, and knee damping.

A healthy human body with a human-like shape and movements is perceived as normal, healthy, and familiar. Also, an exaggerated caricature of a human body and its animated movements can be accepted as somewhat normal and familiar as we expect the caricature to be un-human-like. However, human-like objects, models, robots, or dolls often are designed to mimic normal human body parts, motions, or gestures that almost look normal, but cause an eerie feeling. This psychological reaction to the almost human-like is known as the uncanny valley (Jentsch, 1906; Freud, 1919; Mori, 1970; Eberle, 2012). The uncanny valley can sometimes be described as the perception of something that is familiar, yet incongruous, creating a repulsive effect.

**Figure 1:**
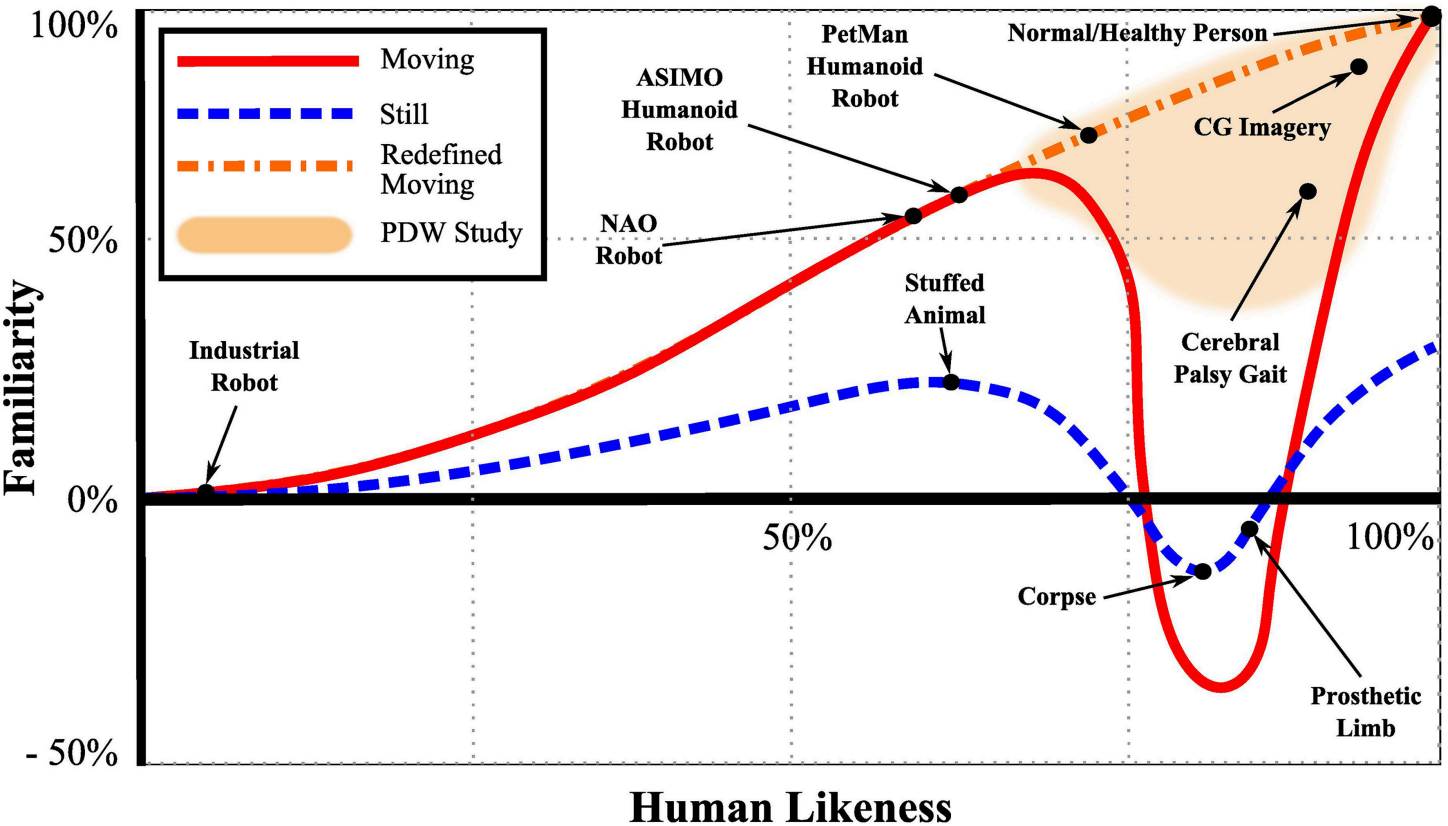
**The Uncanny Valley of still and moving objects**. For still objects, as human likeness increases, so does the observer's familiarity up until a sudden decrease in familiarity into the uncanny valley where familiarity becomes abnormal, perceived with an uneasy or eerie feeling. Initially Mori (1970) proposed the uncanny valley for moving objects, however no study was found to reinforce the concept of the uncanny valley for moving objects. Recent studies with synthetic agents (Thompson et al., 2011; Piwek et al., 2014) show proof that moving objects tend not to fall into the uncanny valley but rather monotonically increase in familiarity with human likeness. The focus of the study presented here is on the right side that relates to what deviations from normal and human-like cause the perception to decrease.

Although the notion of the uncanny valley is widely known, the depths and edges of it are still fuzzy and open for study. It is not clear what changes from normal and human-like will cause one to perceive the altered motions with feelings of uneasiness. As shown in Figure 1, the initial proposal of the uncanny valley is defined as the descent of the plot between human likeness (horizontal axis) and our familiarity (vertical axis) (Mori, 1970).

This decent and the relationship between familiarity and human likeness was initially shown to vary to where moving human-like objects fall further into the uncanny valley than still objects. However, a recent study that examined the existence of the uncanny valley for still and moving human-like objects concluded that, opposed to static human-like characters, augmented human walking movements will not cause any dip in familiarity with increased human likeness. That is, the relationship between familiarity and human-likeness changes monotonically with augmented walking (Piwek et al., 2014). Furthermore, another study by Thompson et al. indicated similar results deducing that when human walking motion parameter changes (joint dis-articulation, jerk, and phase movement changes) are examined, the familiarity rating of a synthetic agent (augmented human motion computer graphic character) by human observers do not show the uncanny valley (Thompson et al., 2011). Although there are studies that verify the uncanny valley for human faces (Seyama and Nagayama, 2007; MacDorman et al., 2009), we were not able not find a clear study that proves the uncanny valley for human body motions. It is interesting to note that one study showed an improvement in familiarity with human likeness in faces with motion compared to still faces (McDonnell et al., 2012).

As we approach the familiarity vs. human-likeness function from the left (low human likeness), we encounter it with lifeless objects, models, and movements such as industrial robots, stuffed puppets, or humanoid robots. The left side of the valley is characterized by motions and attributes that we know not to be human, but have some characteristics that are humanlike. However, approaching this function from the right (high human likeness), that is, coming from the perception of a normal and healthy person, the body motions are highly realistic and match our expectation of how a normal and healthy human typically moves. The top-right side of the valley is populated by very human-like features and motions, however may show some traits that are not exactly normal or healthy. In this article we focus on the right side which is shaded in Figure 1. Specifically, we examine the perception of human walking motions and the limits to which gait will continue to be perceived as normal and human-like in the presence of abnormalities.

Our hypothesis is that gait can appear human-like even when it deviates from perfect temporal and spatial symmetry. Although there are distinct kinematic differences between walking in tennis shoes and high-heeled shoes (Hansen and Childress, 2004), both exhibit a healthy familiar human-like gait. Contrarily, walking with a badly sprained ankle is quickly noticed as a limping gait. Uncanniness emerges when a motion or appearance is close, but not exactly as expected, similar to the feelings that arise when one views individuals walking with a severe injury or disability (Lipson and Rogers, 2000; Henderson and Bryan, 2004). The focus of this study is on the motions that constitute the gait and how to reduce the perception that a gait pattern is abnormal. These results could guide physical therapists in their treatments and would benefit individuals with disabilities that affect gait by determining the gait patterns that minimize the perception that their gait is impaired. Appearance is a major concern for individuals with a disability (Bohannon et al., 1988, 1991). That is, an individual may have the functional ability to walk and it is important for them to be perceived as normal as possible.

## 2. Background

### 2.1. Human gait

**Figure 2:**
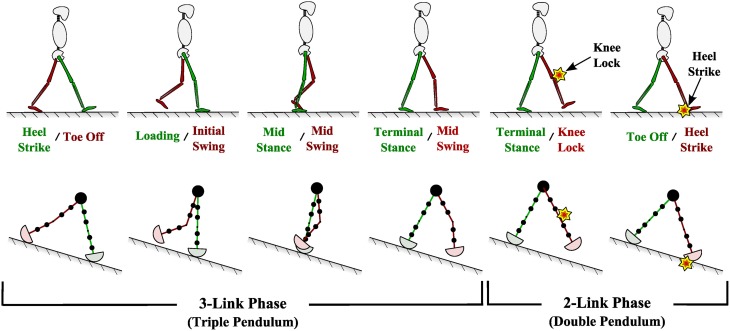
**(Top) Phases of normal and healthy human walking**. **(Bottom)** Passive dynamic walking computational model used in this study to generate various walking patterns.

To ensure understanding of the gait deviations described throughout this paper, we will provide a short background on normal and impaired gait patterns. Normal walking in healthy and unimpaired individuals is smooth and combines complex balancing, shock absorbing, and propelling dynamics along with central nervous system signals to generate efficient locomotion. In a healthy gait pattern, both legs move symmetrically and mirror all dynamics 180° out of phase. As opposed to running, individuals retain ground contact throughout the gait cycle (Perry, 2010; Whittle, 2012). The repeating gait cycle can be subdivided into two periods (stance and swing), eight phases (heel strike, loading response, mid stance, terminal stance, toe-off, initial swing, mid swing, and terminal swing), or three tasks (weight acceptance, single limb support, and limb swing) (Perry, 2010; Whittle, 2012). Some of these subdivisions of normal gait can be seen in Figure 2. The upper body, which includes head, neck, trunk, and arms, moves along as a unit and is considered the passenger unit to the locomotor system, which consists of the legs (Perry, 2010).

Normal healthy walking is symmetric in left-right step length distance, leg swing time, internal joint forces, and external ground reaction forces. The concept of gait symmetry in able-bodied human beings is still an on-going debate (Sadeghi et al., 2000). While many studies exist that assume gait symmetry for the sake of simplicity in data collection analysis, other studies assume gait symmetries if no statistically significant differences are noted on parameters (kinematics or kinetics) measured between limbs. Most able-bodied individuals inherently have some small and unnoticeable spatial and temporal gait asymmetries due to limb dominance or frequent and demanding movements such as in sports (Sadeghi et al., 2000).

**Figure 3:**
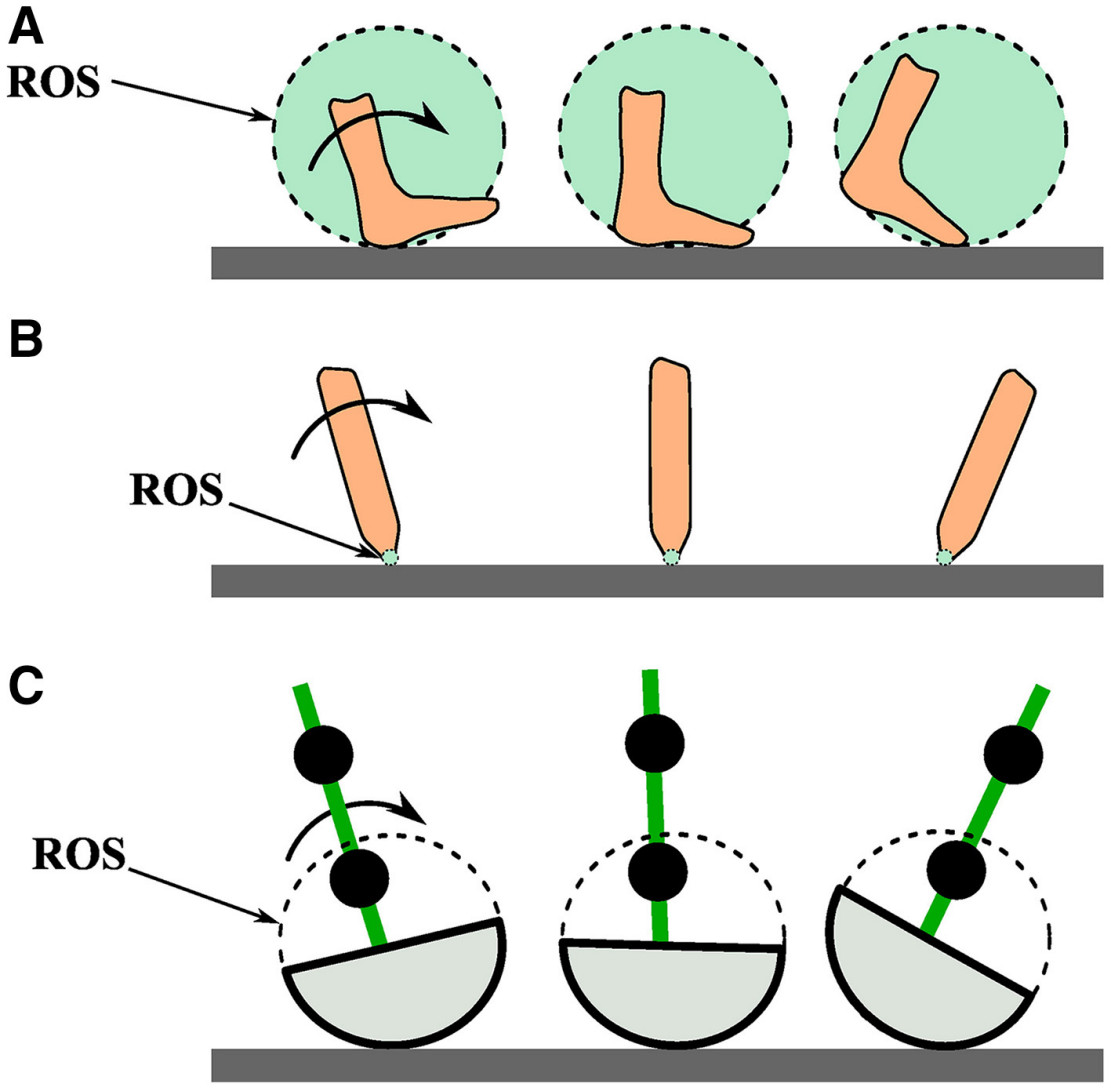
**(A)** A person's foot rolling over the path of its roll-over shape (ROS). **(B)** A point contact rolling over its roll-over shape. **(C)** This study's passive dynamic walker foot rolling over its roll-over shape.

An important aspect of gait is the roll-over shape (ROS) that the foot effectively follows when completing the stance phase during the gait cycle. ROSs of a healthy person during stance phase is presented in Figure 3. ROS have enormous effects on gait kinematics, kinetics, and balance (Menant et al., 2009), and ROS are important in prosthetic design (Hansen et al., 2000; Curtze et al., 2009; Hansen and Wang, 2010). The forces exerted on a foot or by a prosthetic leg onto an individual can be manipulated if the ROS is modified properly (Rietman et al., 2002).

Gait pathology can come in various forms such as deformity, muscle weakness, sensory loss, pain, and impaired motor control caused by disease, injury, or genetic birth traits (Perry, 2010). Such gait pathologies can cause mild or severe gait dynamics or ROS deviations, which may or may not be easily recognizable by other individuals. Deviations from normal walking is often accompanied by compensatory leg dynamics, which may be damaging to other parts of the body. For instance, a person wearing a leg prosthetic which is geometrically identical to the opposite healthy limb, may exhibit recognizable compensatory dynamics such as asymmetric step length, swing time, internal force, or foot ROS asymmetries (Schmalz et al., 2002; Rabufetti et al., 2005; Curtze et al., 2009). ROS for healthy humans can be approximated to be of constant radius and one-third the length of the leg (McGeer, 1990; Adamczyk et al., 2006).

### 2.2. Perception of gait

Humans are very effective at recognizing other humans and the complex motions exerted by other humans (Kozlowski and Cutting, 1977; Loula et al., 2005). While this perception has been generally studied (Blake and Shiffrar, 2007), the creating of the gait perception stimuli has varied. By walking on an asymmetric split-belt treadmill, it has also been shown that humans are able to recognize gait asymmetry in their own gait when walking asymmetry exceeded a specific threshold (Lauzi&re et al., 2014). The gait parameter that corresponded the most with belt speed asymmetry was found to be stance time.

While other forms of methods to recreate human motion for perception analysis has been studied in the past such as PL animation of biological motion (Lee et al., 2002), motion capture (Knoblich and Flach, 2001), or morphing of bipedal locomotion movements (Giese and Lappe, 2002), no study exists that uses a purely dynamics model to evaluate the perception of human gait by systematically altering such a dynamics gait model. This study aims to use a modeled biped model as a perception stimuli by systematically altering the model's dynamics by manipulating its parameters.

### 2.3. Uncanny valley and pathological gait

Humans are keenly aware of walking motions that are close, but not exactly the same as a human makes. To other human observers, a normal healthy gait does not draw any attention and is usually dismissed as ordinary. However, as normal and healthy walking becomes unhealthy or impaired, it starts to raise attention and sometimes uneasy feelings, hence sometimes raising uncanny (eerie) feelings toward the gait mechanics. At an extreme end, this uncanny feeling can be provoked when observing the gait of extremely walking-impaired individuals suffering from neurological movement disorders such as athetoid cerebral palsy or dystonia, resulting in involuntary muscle contractions, repetitive movements, or abnormal postures. However, even smaller alterations from normal healthy gait may be easily recognizable and viewed as abnormal or unfamiliar. Pathological human gait, such as a slightly limping leg or sprained ankle, can be viewed as human-like and normal, yet the impairment will be quickly identified.

In healthy humans, the two sides of the body are mostly symmetric with regards to mass and strength; thus, it makes biomechanical sense to have both knees at the same location. However, when wearing a transfemoral prosthesis, the mass and strength of the two legs are no longer equal and the biomechanical reasons to keep the same prosthetic knee location no longer exist. Moving the knee location adds a degree of freedom in the prosthesis design process that allows the gait dynamics to be adjusted to a desired gait pattern (Sushko et al., 2012). However, changing the knee location depends on the answer to an essential question for this study: what amount of knee location asymmetry can be considered normal or human-like? Note that we are only concerned with the bio-mechanical movements of leg limbs and how these movements are perceived in this study. We are not investigating the effects of limb thickness or texture perception, such as wearing a Flex-Foot Cheetah prosthetic blade foot (Grabowski et al., 2010).

### 2.4. Uncanny valley and artificial gait

Toyota's ASIMO (Sakagami et al., 2002) and Aldebaran Robotics's NAO (Anderson et al., 2011) robots are statically stable robots that are able to simulate a slow and careful walking pattern while always keeping their center of gravity above their support base. Humans can walk this way, but rarely do. Such statically stable robotic gait is only partially perceived as human-like and can come off as stiff, “robotic,” and sometimes uncanny. While more proficient in its gait, Boston Dynamics's PetMan (Raibert, 2010) is an anthropomorphically correct biped able to mimic gait very similar to humans. PetMan is able to skillfully navigate across obstacles such as stairs and withstand moderate perturbations during gait. Nonetheless, its more realistic motions invoke an unhuman-like perception of its movements. These humanoid robots are perceived to be on the left side of the uncanny valley and so are of little direct interest to our study and hypothesis about the right side of the valley.

On the other hand, dynamically stable walking robots such as a passive dynamic walker (PDW), exhibit a more fluent and human-like gait. A PDW is a biped walking robot that walks down a decline with gravitational energy as its only source of power and with no active feedback (McGeer, 1990). PDW gait is shown to be kinematically and kinetically similar to human gait (Adamczyk et al., 2006; Kuo, 2007; Handžić and Reed, 2013a,b. While PDWs can be used to recreate and analyze normal and pathological human walking patterns, they can also be utilized to study the effects on gait caused by manipulating swinging limb parameters such as leg lengths, leg masses, joint stiffness, or ROS (Honeycutt et al., 2011).

## 3. Materials and methods

### 3.1. Passive dynamic walking gait

This study employed a PDW computational model because the PDW model is repeatable, precise, and can be systematically altered in order to implement altered gait patterns. This consistency allows the controlled variation of desired parameters (i.e., step length, limb mass, joint stiffness, ROS etc.) without the inconsistency of human sensorimotor control under the same walking conditions.

The PDW model is a two dimensional nine-mass multi-pendulum system with constant-radius-shaped feet. That is, it represents an anthropomorphically correct walking human from the waist down and viewed from a two dimensional sagital plane. PDW masses are represented as one hip mass and two masses per each thigh and shank. The PDW model also rolls over a constant radius roll-over shape just as a walking human would (Figure 3C). Just as in human gait, the PDW legs progress through two distinct phases, stance and swing, as it advances down a decline as seen in Figure 2. During a step and before knee lock, the PDW is modeled as an inverted triple pendulum as the shank swings forward, after which it turns into an inverted double pendulum. The kinematics of our PDW can be derived with the Lagrangian formulation, while the knee lock and heel strike collision events can be described with conservation of angular momentum. The mathematical modeling for our PDW with point feet can be reviewed in McGeer (1990), Chen (2005), and Honeycutt et al. (2011). Although, the PDW can walk down a greater decline, our model walks down a slope of 3.5° for all gait variations presented in this study. We specified the PDW model height, thigh length, and shank length, mass and mass distribution according to widely surveyed anthropomorphic body segment data (Drillis et al., 1964). The roll-over shape for normal walking was taken to be one-third leg length as found in Adamczyk et al. (2006). All PDW deviations presented in this study were stable for at least fifty steps.

### 3.2. Measured normal gait

In addition to the systematically altered gait patterns derived from the PDW modeled gait, one gait pattern was collected from a healthy individual walking at a comfortable speed over level ground. The individual was 28 years of age, 93 kg (205 lb), and was 1.85 m (6 ft 1 in) tall. The individual walked barefoot on a stationary treadmill at 0.8 m/s. The treadmill and the motion tracker system are part of the Computer Assisted Rehabilitation Environment (CAREN) system. The gait was recorded using a VICON® motion capture system with ten Bonita B10 cameras set to record at 100 Hertz. Reflective markers (14 mm in diameter) were placed on both left and right hip (anterior superior iliac spine), knee joint, ankle joint, and big toe (phalanges). The individual walked for ten strides at steady state and an average of those motions was used as the comparison video. The individual whose gait was recorded signed a University of South Florida Institutional Review Board (IRB) consent form before volunteering to be analyzed for this study.

### 3.3. Passive dynamic walking animation videos

**Figure 4:**
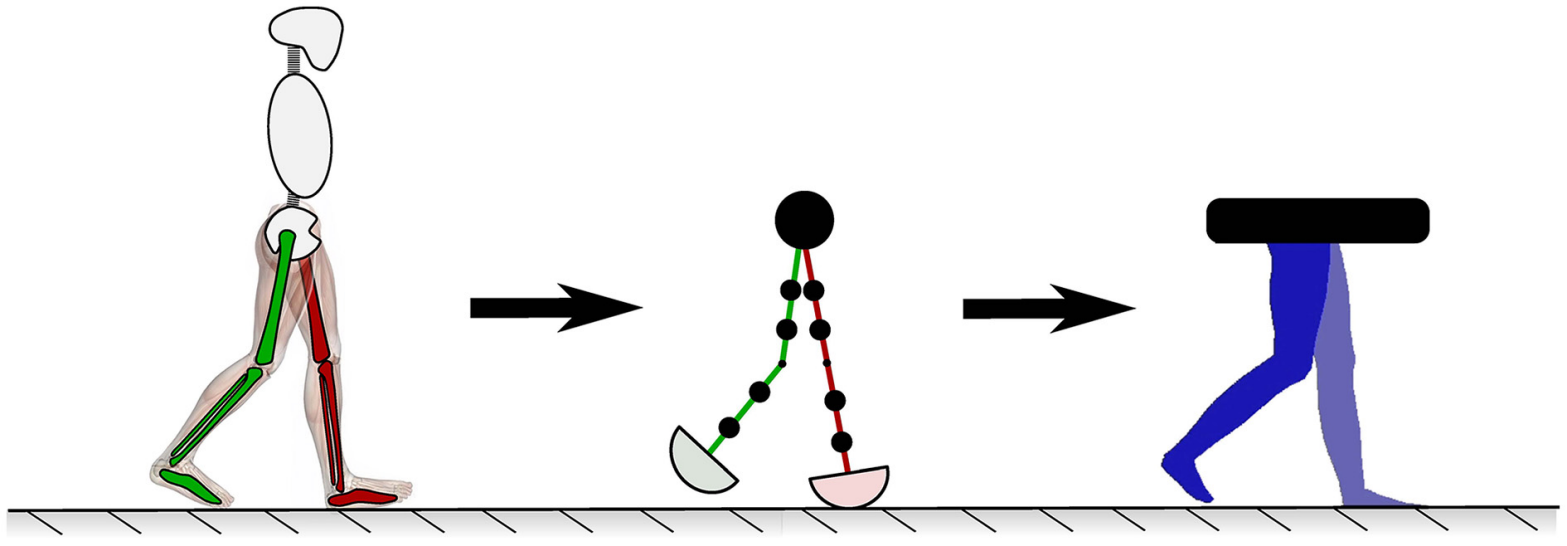
**The human lower limb model presented to participants was modeled with a PDW to move naturally while being carefully depicted to look like normal human limbs**.

Because this study predominantly focuses on normal and abnormal human walking motions, the PDW model closely depicts the aesthetics of a person walking when viewed from the side (silhouette). This helps to increase the participant's familiarity and human likeness of the presented walking models. The animation silhouette was closely depicted to mimic human muscles, joints, knees, and feet by considering waist, mid-thigh, and max calf circumference as outlined by the United States Department of Health and Human Services Health Statistics Report (McDowell et al., 2008). This aesthetic transformation of our PDW model can be seen in Figure 4. Note that the focus of this study is on the motions of the gait and not the static appearance of the legs. Although the PDW walks down a decline, it was rotated to look as if it is walking on level ground. Feet were animated by interpolating the foot angle trajectory of the actual recorded normal gait and fitting it onto the computational dynamics of the PDW model since the PDW model does not simulate feet.

While previous uncanny valley studies that analyzed body motions used computer generated animations controlled by deteriorated and augmented human body motions (Piwek et al., 2014; Thompson et al., 2011), the gait motions in this study are based on computational dynamics allowing systematic and precise augmentation or leg kinematics.

Various PDW walking parameters were computationally and systematically varied to deliberately deviate from familiar and human-like (normal) gait to explore the perception of impairment and uncanniness of human gait. Although the PDW computational model can simulate many parameters with any parameter resolution, that would yield many videos to be judged by participants, which would result in a prolonged experiment per participant. Table 1 shows the different parameter categories chosen for this study that were presented to the participants. All PDW leg variations were applied to the leg closest to the observer (i.e., darker, right). Note that Equation (1) is used to define percent asymmetry between two parameters. Equation (1) is used to define percent asymmetry for all parameters. The negative values in Table 1 for knee height refer to a decrease in knee location and the negative values in gait cadence refer to a slower speed.

(1) $$Asymmetry\;(\%)=\left(\frac{abs(Left-Right)}{(Left+Right)/2}\right)$$

**Table 1:** **Five PDW parameter categories were studied**.

**Gait cadence (%)**	**Knee height asymmetry (%)**	**Spatial and temporal asymmetry (%)**	**ROS asymmetry (%)**	**Knee damping with mass asymmetry (%)**
−50	+83	5 (LaTa)	29	0
−25	+57	13 (LsTa)	66	40
+25	+22	5 (LaTs)	100	100
+50	−26	13 (LaTs)		118
	−40	5 (LaTa)		
	−61	13 (LaTa)		

*The 23 listed here, plus recorded and PDW modeled normal videos were presented. L = Step Length, T = Swing Time, s = Symmetry, a = Asymmetry*.

### 3.4. Gait videos

The following videos were presented to participants. Participants judged all videos on the the basis of two separate metrics: impairment and uncanniness.

#### 3.4.1. Measured normal gait

This gait pattern was recorded from a healthy individual who had no asymmetries or abnormalities. The recorded gait cadence was measured at 80 steps/min. This video was included to compare the perception of the PDW modeled normal gait to a human gait. Note that the measured normal gait from this human participant has an approximately 25% slower cadence than the modeled normal gait. This difference highlights the benefit of the PDW model for allowing a systematic alteration of the gait patterns; we cannot impose a specific change in a human, but can in the PDW modeled system.

#### 3.4.2. PDW modeled normal gait

The normal PDW modeled walking pattern was perfectly symmetric between left and right sides. This normal gait walking cadence was matched to that of a healthy adult walking cadence at 110 steps/min (Perry, 2010). This video was shown as a baseline and for comparison to a recorded walking pattern from a healthy human participant. This video was also used as the stimulus (base) for comparison in each category.

#### 3.4.3. Category 1: gait cadence

Gait cadence may affect the observer's perception of the gait, so four different videos of the PDW modeled normal gait at four different speeds were included in the study (two slower and two faster) (−50, −25, +25, and +50%).

#### 3.4.4. Category 2: knee height

**Figure 5:**
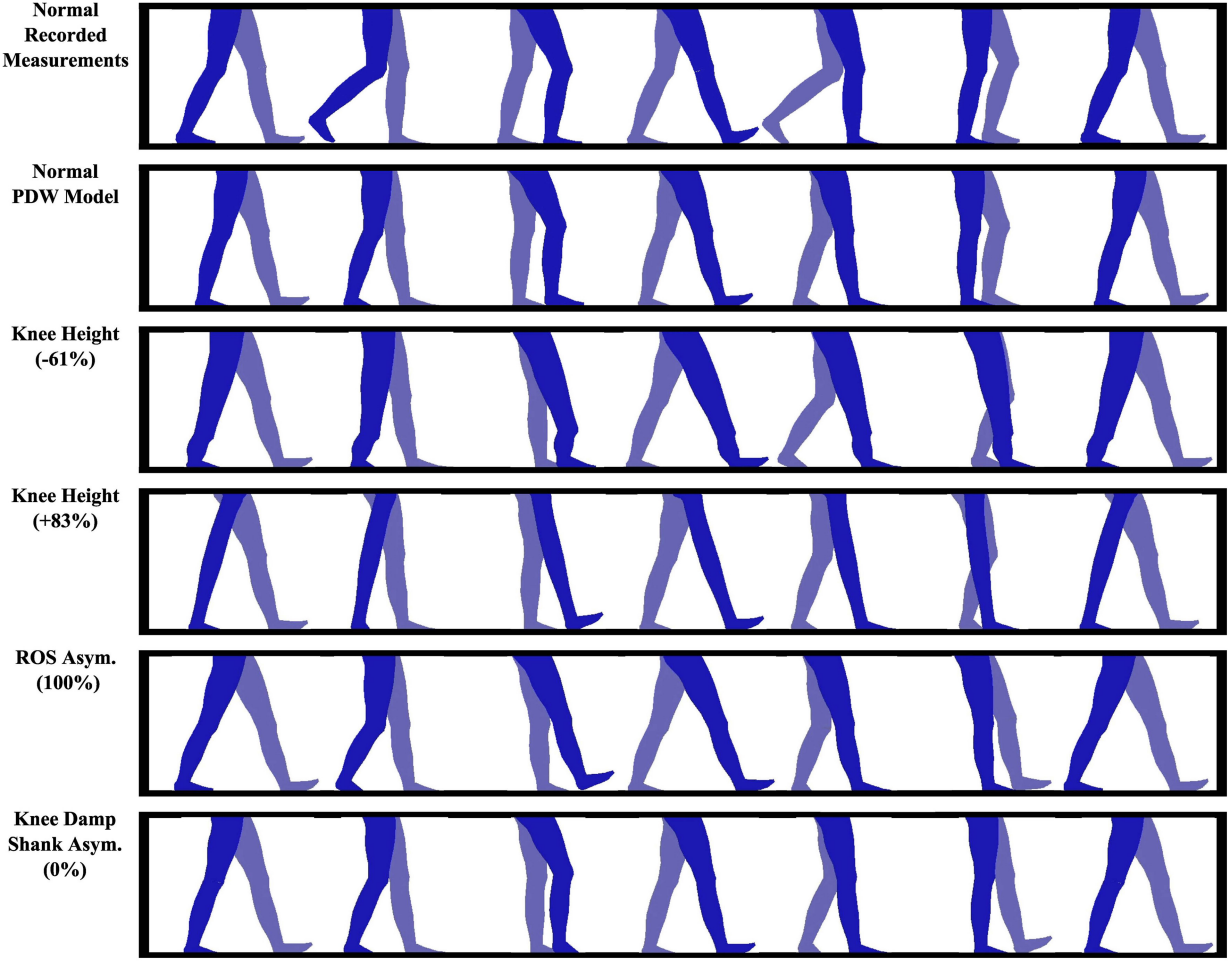
**Some of the passive dynamic walker models that were presented to participants**. All videos are included in the Supplementary Material.

As previously reviewed in the background section, prosthetic knee location (knee height) may be altered in order to gain spatial, temporal, kinetic symmetry, or comfort while walking. These alterations aim to determine how much deviation in knee height symmetry is noticeable and perceived as uncanny. As listed in Table 1, we present three videos where the walking model has a knee asymmetry with one knee raised and three videos that show the walking model with knee asymmetry by lowering one knee. All models in this category have symmetric step lengths and swing times. Because the knee is displaced very close to the hip, the video with +83% knee height shows no knee, as seen in Figure 5, but is present in the other videos. Knee heights are not evenly distributed from symmetric knee position because equal changes above and below the knee did not yield a stable PDW.

#### 3.4.5. Category 3: spatial and temporal asymmetry

In this video set, our intent is to examine if spatial and temporal asymmetries such as caused by limping, partial leg paralysis (hemiplegia), or a leg prosthesis will be noticeable, that is, viewed as abnormal or uncanny. In two videos, step length is held symmetric while swing time asymmetry is created (LsTa), in two videos swing time is held symmetric while step length asymmetry is created (LaTs), and in another two videos equal amounts of step length and swing time asymmetries were created (LaTa).

#### 3.4.6. Category 4: ROS asymmetry

Walking impairment and some prosthetics can cause asymmetries in foot roll-over shape (ROS). We included three different walking patterns with asymmetric ROS foot curves. At no ROS asymmetry, both ROS are 0.333 m (1.09 feet) in radius, whereas at 100% ROS asymmetry the left ROS is 0.333 m (1.09 feet) while the right ROS is 0.111 m (0.36 feet).

#### 3.4.7. Category 5: knee damping with asymmetric shank mass

**Figure 6:**
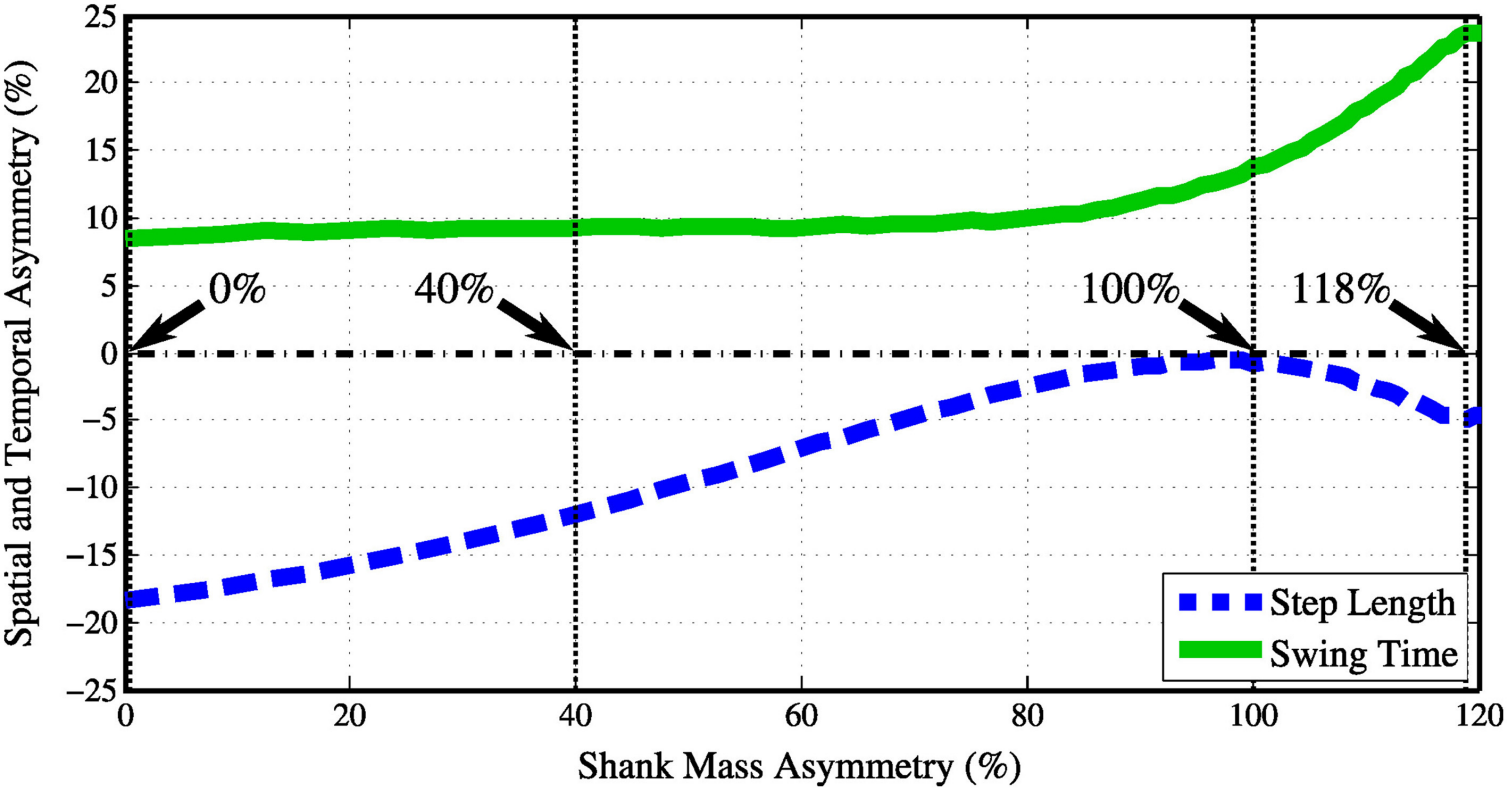
**As the right knee was damped with 0.275 Newton-radians the shank asymmetry was increased from 0 to 120%**. The step length and swing time asymmetries are on opposite legs.

Four videos are included that model damping in the right knee, which simulates a stroke gait. To compensate for the damping, four different PDW shank masses were tested. The intent was to examine if a damped (i.e., impaired, injured, damaged) knee is recognizable or abnormal. If asymmetry with a damped knee is recognizable, is it possible to remove the uncanny effect by altering the impaired gait? We attempt to alter the damped gait by imposing a shank mass asymmetry. The kinematic effects on spatial and temporal gait asymmetry can be viewed in Figure 6. Four videos were recorded at 0, 40, 100, and 118% shank mass asymmetry. The knee damping was chosen to be 0.275 Newton-radians, which was the highest knee damping value that allowed a stable gait pattern in the PDW.

### 3.5. Experimental setup and protocol

Data collection was completed using a custom internet website. This straightforward website presents one gait video (Section 3.4) at a time, while users are able to rate the shown videos on the gait's impairment and uncanniness. The website cycles through all 25 gait videos that are shown in Table 1 in a random order. The 25 videos consist of the recorded and PDW modeled normal walking pattern, and the 23 videos of altered gait patterns using the PDW described in Table 1.

While watching each walking video, participants answered two questions which were presented on the screen simultaneously, each on a 7-point Likert scale (Likert, 1932) for that video. The first question asked the participants to discretely rate the video on the impairment of the gait, asking *“How normal and unimpaired does this gait appear?”*. That is, participants were asked to judge the presented videos with seven options ranging from *“Normal”* to *“Very abnormal or impaired,”* with *“A little abnormal or impaired”* at the halfway point. Similarly, the second question asked the participants to rate the shown video on the uncanniness of the gait, reading *“How eerie or uncanny does this gait appear?”*. The participants were given as much time as they wanted to evaluate each video which cycled from beginning to end indefinitely. The duration of all the videos was roughly thirty seconds long, however slightly varied in length depending on gait speed.

The participants are asked to perform at least 25 video ratings, however some participants completed as many as 144 video ratings with a median of 70 video ratings. A total of 1582 video ratings were submitted by 42 participants, however, to improve consistency, only participants that rated 4 or more gait videos were considered, which yielded 33 valid participants. Furthermore, if a video was rated twice by a participant, only the first perception score was included, reducing a potential bias from individuals that rated a video multiple times. Each video was rated a minimum of 26 times with a median of 61 video ratings per gait video. All of the videos shown to the participants are included as Supplementary Material.

The web page includes simple instructions and a clear link to an approved minimal risk University of South Florida Institutional Review Board (IRB) consent form with a waiver of documentation of consent.

### 3.6. Statistical analysis and evaluation

Participants rated walking videos on a symmetric 7-point Likert scale. Because independent participants evaluate the walking videos and the ranked quantitative responses hold true throughout the Likert scale range, we assume a continuous linearity between Likert scale points and treat the acquired data as ordinal interval-level. A Chi-square goodness-of-fit test revealed that the comprehensive data does not follow a normal distribution [χ^2^(_6, *N* = 33_) = 844, *p* < 0.001]. Data within each category was also found not to follow a normal distribution, where the statistics of each video category Chi-squared will be included in the following results section. Because the data for each category of videos does not follow a normal distribution, we use a Kruskal-Wallis one-way analysis of variance non-parametric test to verify whether video ratings within each category of videos originated from the same distribution (i.e., are they statistically significantly the same). A Kruskal-Wallis one-way analysis of variance by ranks test (a.k.a. Kruskal-Wallis H test or Dunn's test) is a rank-based nonparametric multiple comparison test. This *post-hoc* test is used to determine if there are statistically significant differences between two or more videos in each video category rated on the 7-point Likert scale.

The base/control gait perception rating for this experiment is the measured normal walking pattern. Before we are able to asses judgment on the impairment and uncanniness of all the videos, we first set out to compare our PDW modeled normal gait to the measured gait and determine if participants viewed our modeled walking pattern as being as normal as the recorded gait. To evaluate the statistical significance between the recorded and normal walking video, we apply a Wilcoxon rank-sum test used for nonparametric testing of the null hypothesis that the two compared populations stem from the same population. This test will be used to evaluate how close to the actual recorded normal gait the PDW modeled normal gait is.

## 4. Results

### 4.1. Perception of impairment

**Figure 7:**
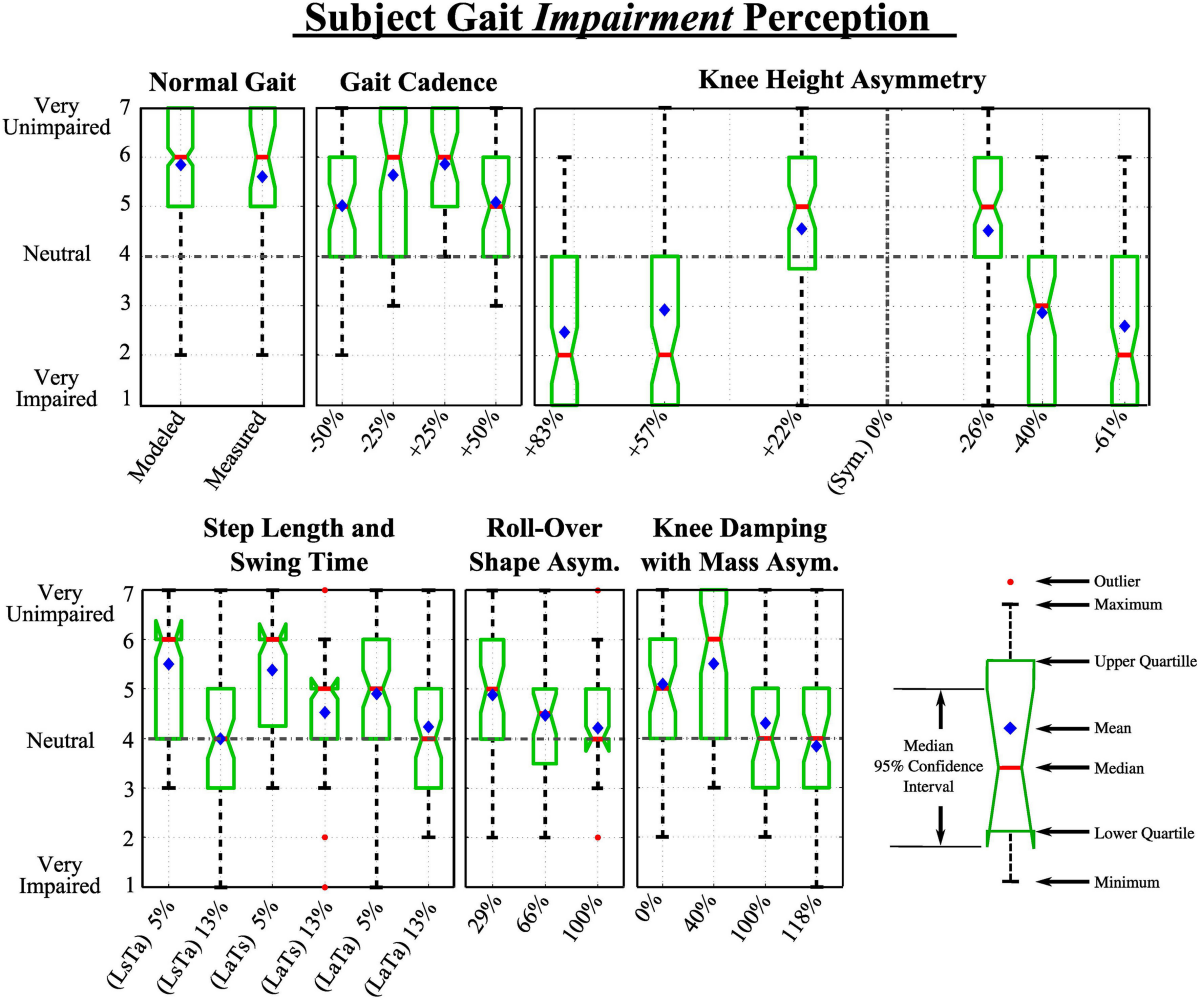
**Extended box and whiskers notch plots show participant's responses to videos in each category in response to the question: *“How normal and unimpaired does this gait appear?”***.

In this section, The perception of all 25 gaits in terms of gait impairments was analyzed, that is, participants' perception of the gaits' pathological nature. The results of each category are shown in Figure 7. All videos in each category were compared to normal gait, that is, comparison statistics included PDW modeled gait perception results for each category.

#### 4.1.1. Normal gait

A Wilcoxon rank-sum test was used to evaluate the difference in the responses of our 7-Likert scale question on gait impairment. We found a non-significant effect between the two data sets, thus no statistically significant difference was found. The mean ranks of the recorded and modeled gait data sets were 142 and 162, respectively; *Z* = 1.12, *p* > 0.05. The number of collected ratings for the recorded and PDW modeled gait pattern was *n*1 = 26 and *n*2 = 294, respectively. The medians of the recorded and modeled data were both 6 as seen in Figure 7.

#### 4.1.2. Category 1: gait cadence

Chi-squared goodness of fit analysis for this category revealed that the data did not follow a normal distribution [χ^2^_(6, *N* = 33)_ = 400, *p* < 0.001]. Nonparametric Kruskal-Wallis one-way analysis of Variances showed statistically significant difference between the perceived abnormality due to impairment of different gait cadence [*H*_(4, 466)_ = 24.4, *p* < 0.001]. *Post hoc* analysis showed that participants were able to spot that there was something abnormal and altered between the modeled normal walking pattern and a gait that is 50 and 50% faster. However, participants were not able to statistically significantly distinguish the normal from gaits slowed down 25% and sped up 25% within this category.

#### 4.1.3. Category 2: knee height

Data sets in this category were found to not follow a normal distribution [χ^2^_(6, *N* = 33)_ = 317, *p* < 0.001]. A statistically significant difference was detected [*H*_(6, 686)_ = 327.6, *p* < 0.001]. Participants perceived all presented knee location changes as statistically significantly different compared to the normal gait. Participants evaluated knee heights of +83, +57, −40, and −61% as noticeably and highly abnormal or impaired, measuring their median, averages, and confidence intervals below neutral (4). Knee heights of +22 and −26% were only perceived as moderately impaired, which indicates that some knee height asymmetry with spatial and temporal gait symmetry could be dismissed as somewhat normal by observers. Participants were slightly more consistent in rating a low knee height as abnormal compared to higher knee locations (based on the confidence interval range).

#### 4.1.4. Category 3: spatial and temporal asymmetry

Data sets in this category were found to not follow a normal distribution [χ^2^_(6, *N* = 33)_ = 397, *p* < 0.001]. A statistically significant difference was found within this category group [*H*_(6, 689)_ = 146, *p* < 0.001]. *Post-hoc* analysis revealed both step length (L) and swing time (T) left-right asymmetries produced statistically significant differences compared to normal gait when a 13% asymmetry was imposed, however at 5% asymmetry the gait was not perceived as impaired. That is, participants did not see small independent changes in swing time and step length as impaired. The gait was perceived as recognizably impaired at 13% step length asymmetry (LaTs) (mean rank = 238), while being perceived as yet more impaired at 13% swing time asymmetry (LsTa) (mean rank = 193). However, the difference in impairment perception between these two videos was not statistically significantly different (Wilcoxon *Z* = 0.55, *p* = 0.58).

#### 4.1.5. Category 4: ROS asymmetry

The data sets in this category did not follow a normal distribution [χ^2^_(6, *N* = 33)_ = 342, *p* < 0.001], while a statistically significant difference among videos in this category was found [*H*_(3, 401)_ = 68, *p* < 0.001]. Post hoc analysis showed participants perceived all videos in this category with a statistically significant difference compared to the normal gait. Walking videos with 29, 66, and 100% ROS asymmetry were perceived as minimally (mean rank = 160), moderately (mean rank = 127), and highly impaired (mean rank = 102), respectively.

#### 4.1.6. Category 5: knee damping with asymmetric shank mass

The data sets in this category did not follow a normal distribution [χ^2^_(6, *N* = 33)_ = 262, *p* < 0.001], while a statistically significant difference among videos in this category was found [*H*_(4, 462)_ = 89, *p* < 0.001]. Participants perceived all but one (40%) shank asymmetry with knee damping videos in this category with a statistically significant difference compared to the normal gait, seen in Figure 6. Although the 40 and 100% shank mass asymmetry had similar temporal asymmetries, 9.2 and 13%, receptively, only the 100% shank asymmetry was perceived as significantly different from normal gait. However, this may be caused by the spatial asymmetry in gait, which was 12 and 0% for the two videos respectively. Once the temporal asymmetry increased to 24% with a 4.5% spatial asymmetry, the perception of impairment was at its maximum.

### 4.2. Perception of uncanniness

**Figure 8:**
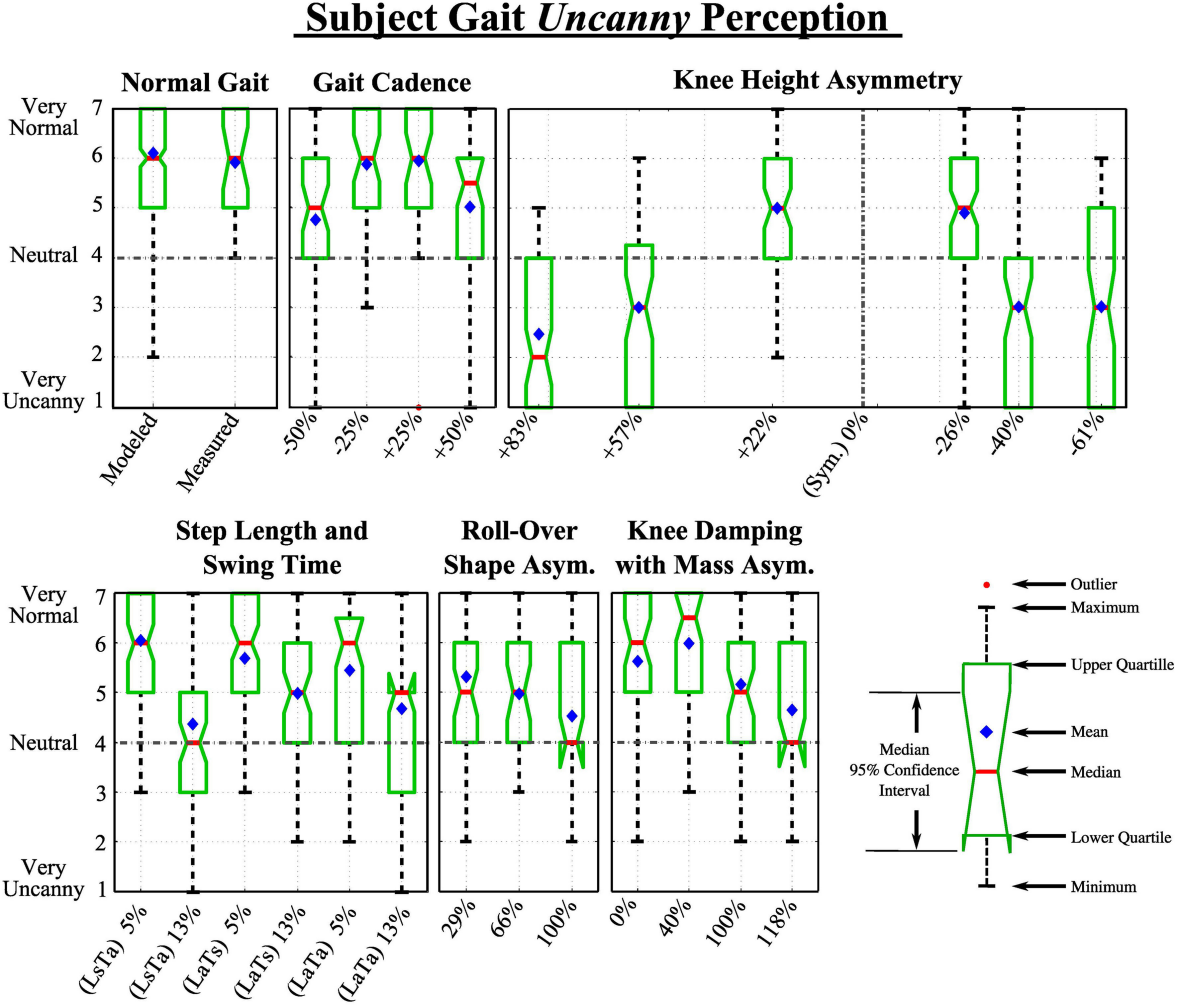
**Extended box and whiskers notch plots show participant's responses to videos in each category in response to the question: *“How eerie or uncanny does this gait appear?”***.

Here the results of participants' perception of 25 gaits in terms of how uncanny (eerie or strange) the gaits appear are presented. The results for this second metric for each category are shown in Figure 8.

#### 4.2.1. Normal gait

As with the impaired perception metric, we initially compare a measured and PDW modeled normal walking pattern. Wilcoxon rank-sum test yielded a non-significant effect between the two data sets, thus no statistically significant difference was found. The mean ranks of the recorded and modeled gait data sets were 142 and 162, respectively; *Z* = 1.10, *p* > 0.05. The number of collected ratings for the recorded and PDW modeled gait pattern was *n*1 = 26 and *n*2 = 294, respectively. The perception rating medians of the uncanniness of the recorded gait was 6, while the median rating for the modeled normal video was also 6.

#### 4.2.2. Category 1: gait cadence

The participant rating data did not follow a normal distribution [χ^2^_(6, *N* = 33)_ = 214, *p* < 0.001]. We found a statistically significant difference between the perceived abnormality due to impairment of different gait cadence [*H*_(4, 466)_ = 47, *p* < 0.001]. Participants found something more uncanny about the gait that was 50% slower and 50% faster, however median Likert score for both these altered gaits was 5 and 5.5, respectively. This shows that participants saw a slight uncanniness compared to the normal modeled walking pattern, but could not definitely say that it was uncanny. Participants were not able to statistically significantly distinguish the normal from gaits slowed down 25% and sped up 25% within this category.

#### 4.2.3. Category 2: knee height

Data sets in this category were found to not follow a normal distribution [χ^2^_(6, *N* = 33)_ = 317, *p* < 0.001]. Among them a statistically significant difference was detected [*H*_(6, 686)_ = 327.6, *p* < 0.001]. Participants perceived all presented knee location changes as statistically significantly different compared to the normal gait. In other words, individuals rated all deviations from normal gait as uncanny to some degree.

Participants evaluated knee heights of +83, +57, −40, and −61% as noticeably uncanny, measuring their median, averages, and confidence intervals below the neutral score of 4, however knee heights of +22 and −26% were only perceived as moderately impaired and below a neutral uncanny perception. These results are very similar to the previously discussed impairment ratings with the exception of knee height asymmetry of +57 and −40% which was rated a 3 instead of a 2.

#### 4.2.4. Category 3: spatial and temporal asymmetry

Data sets in this category were found to not follow a normal distribution [χ^2^_(6, *N* = 33)_ = 329, *p* < 0.001]. A statistically significant difference was found within this category group [*H*_(6, 689)_ = 135, *p* < 0.001]. As opposed to the impairment ratings, uncanny ratings were consistently lower, however the same trends persisted. Both step length (L) and swing time (T) left-right asymmetries produced statistically significant differences in uncanniness compared to normal gait when a 13% asymmetry was imposed, however at 5% asymmetry the gait was not perceived as impaired. That is, participants did not see small independent changes in swing time and step length as uncanny, while larger temporal and spatial asymmetries each at 13% were perceived as recognizably more more uncanny at medium ranks at 4 and 5, respectively.

#### 4.2.5. Category 4: ROS asymmetry

With all the previous groups, the collected ratings for this category was a normal distribution [χ^2^_(6, *N* = 33)_ = 321, *p* < 0.001], while a statistically significant difference among videos in this category was found [*H*_(3, 401)_ = 66, *p* < 0.001]. As with the gait impairment perception, post hoc analysis showed participants perceived all videos in this category with a statistically significant difference compared to the normal gait. Walking videos with 29, 66, and 100% ROS asymmetry were perceived as minimally (mean rank = 163), moderately (mean rank = 132), and highly impaired (mean rank = 100), respectively.

#### 4.2.6. Category 5: knee damping with asymmetric shank mass

Again, the data sets in this category did not follow a normal distribution [χ^2^_(6, *N* = 33)_ = 375, *p* < 0.001], while a statistically significant difference among videos in this category was found [*H*_(4, 462)_ = 55, *p* < 0.001]. Participants perceived all but two (0 and 40%) shank asymmetry with knee damping videos in this category with a statistically significant difference compared to the normal gait. As seen in Figure 6, the same trends arise as in the impairment rating results, with the exception that 0% shank mass asymmetry with knee damping was not significantly different then a normal walking pattern.

## 5. Discussion

Statistically, participants were shown not to be able to effectively differentiate between a recorded healthy human gait and a modeled PDW walking pattern in terms of impairment and uncanniness. Hence, it was viable to compare a modeled PDW gait to further walking models that have been systematically altered. This also suggests that there are significant visual characteristics of PDW gaits that are similar to human gaits as is expected since the kinematics are similar (Donelan et al., 2002; Handžić and Reed 2013b). Although the trend was similar to impairment ratings, uncanny rating confidence intervals were generally shifted slightly toward normal perception.

Trends of the perception on gait impairment is similar to the perception on gait uncanniness, however the uncanny perception seems generally slightly and consistently closer to normal PDW gait than the impairment perception of the same walking pattern. This means that the participants consistently recognized abnormal gaits as pathological, but did not feel an equally strong uncanny or eerie feeling while watching the gait. Thus, we believe most of the perceptions in this study are along the top-right portion of the human-likeness/familiarity shown in Figure 1.

The combined results of this study confirms the conclusions drawn by Thompson et al. (2011) and Piwek et al. (2014), which strengthen the counterclaims against an uncanny valley for computer generated synthetic human body motions. Similar to their results, we were only able to find monotonically decreasing familiarity with heightened abnormality. However, we can only speculate about why. Our abnormality (stimuli) resolution could have been too low to find a valley dip. In addition, the amplitude of the imposed abnormality may not have been substantial enough to map it onto the most right side of the uncanny valley. Furthermore, our study only focused on the lower extremity kinematics, hence in the light of this focus and previous studies, the effects of the uncanny valley may be minimal or even nonexistent.

Normal gait with a gait cadence increased or decreased by 50% was noticed as slightly more impaired and uncanny when compared to a normal gait cadence. This may indicate that when seeing someone walking hastily or abnormally slow, it can be interpreted as out of the ordinary and draws attention, signaling that some impairment or abnormalities are present. Although both the impairment and uncanniness of these videos were significantly different than the normal walking pattern, participants' medium rating hovered between 5 and 6, that is, neutral to very unimpaired/uncanny. Such a reaction may draw some attention from observers, however would generally not be considered abnormal.

A inverse “V” pattern shows the increase of participants' gait impairment and uncanniness perception with knee height asymmetry, with a focal area between +10 and −20% knee height change. These results imply that given step length and step time symmetry, some knee height asymmetry can be unrecognizable or even perceived as normal. As opposed to the other categories, alteration of knee height symmetry provoked the highest participant impairment or uncanny ratings. It is shown that the higher the knee location is moved from its symmetric position, the more the gait is perceived as impaired or even uncanny. These results also suggest that a prosthetic design with a lowered knee location for functional improvement (Sushko et al., 2012; Ramakrishnan, 2014) may be unnoticeable to some extent. It should be noted that the experiment did not examine if or how clothing and wearing loose-fitting clothes would help to hide the effect of a prosthetic with a knee location in a different location, but these effects are likely to mask the knee location.

Separately, 5 and 5% LaTs did not produce a perception of impairment or uncanniness with participants, inherently suggesting that some gait asymmetry is not noticeable by observers and it should be noted that healthy individuals are known to have some asymmetric gait parameters (Sadeghi et al., 2000). For example, for observers to consistently not notice gait asymmetry such as a limb caused by a prosthetic or injury, one can walk with a 5% spatial or temporal asymmetry. However, it is interesting to note that 5% simultaneously in both measures produces a moderate perception of abnormality but with the confidence interval below the neutral perception rating. It may be concluded that compounding these asymmetries may cause greater perceptions in abnormality, however this seems not to be the case for 13% LaTa. The 13% LaTa was rated similar to the 13% LaTs, while 13% LsTa was rated more impaired and uncanny than 13% LaTa. A further study using more combinations of these asymmetric gait measures would help to understand the perceptual interactions with gait asymmetry more fully.

Although more ROS asymmetries would clarify a trend, it can be concluded that with all factors symmetric, a ROS asymmetry below around 35% can pass as minimally impaired or uncanny by observers. The trend implies that a ROS asymmetry below 15% may not be distinguishable from a normal and healthy gait. This is not surprising since ROS have enormous effects on gait kinematics, kinetics, and balance (Menant et al., 2009). ROS are important in prosthetic design (Hansen et al., 2000; Curtze et al., 2009) and for reducing forces on the user's stump (Rietman et al., 2002). Specially-designed shoe soles can also benefit individuals with cerebral palsy, Parkinsons, and stroke (Rodriguez and Aruin, 2002).

Category 5 results imply that if a person suffering from an impairment causing damping in a knee (injury, neurological, etc.), that person could be seen as impaired or even slightly uncanny. However, imposing an accompanying asymmetry, such as adding an asymmetric mass distribution, can potentially alleviate the perception of impairment or uncanniness. In other words, as one gait asymmetry is imposed that causes gait perception of impairment and uncanniness, a second gait asymmetry may be applied to some degree to negate these perceptions. This combination of asymmetries could lead to gait patterns that balance the perceptual and dynamic aspects of gait.

Results from Category 5 agree with the conclusions drawn from Category 3 since, looking at Figure 6, it can be concluded that the swing time asymmetry has a greater effect on participants noticing the abnormality than step length asymmetry. Although a person may step with symmetric step distances, the difference in limb swing time is far more noticeable to observers as shown with gait of Category 5 100% shank mass asymmetry and Category 3 13% LsTa. It is interesting to note that these results are comparable to Lauzi&re et al. (2014) who looked into the perception of ones own gait asymmetry (internal), which concluded that the parameter that corresponded the most with belt speed asymmetry was found to be stance time.

This normalizing of the perception of joint damping can also potentially be achieved by altering other gait parameters such as having a foot roll-over shape or knee height asymmetry, however, this is still open for future studies.

## 6. Conclusions and future work

In this study we outlined the boundaries of perceived gait impairment and uncanniness of some pathological or altered gait patterns including moved knee height and asymmetric foot roll-over shape radii. Despite a selected number of gait alteration parameters, we were able to explore the perception of pathological or uncanny gait. Generally, perception rating trends were the same between impaired and uncanny ratings, however the uncanny rating was consistently more normal. This similarity in trends may suggest a coupling between the perception of impairment and the uncanny. Although we have shown that altering human gait parameters alters the perception of normal and healthy walking to observers, further investigation with different types of gait pathologies and a greater resolution of abnormalities in walking patterns for each category is needed.

We conclude that there clearly is a gray and undefined area in human perception in gait, where human gait may be abnormal while being perceived as unimpaired or uncanny. The gait abnormalities that we analyzed were gait cadence, knee height asymmetry, spatial and temporal walking asymmetries, and foot roll-over shape asymmetry. We also examined the perception of gait by changing two independent gait parameters, specifically asymmetric knee stiffness and shank mass asymmetry. This multi-parameter analysis clearly showed that it is possible to alter the perception of a gait impairment by manipulating different gait parameters. These results are promising and such a multi-parameter manipulation technique may be useful in the field of prosthetic or hemiplegic gait analysis and rehabilitation, in that a noticeable gait asymmetry could be hidden by imposing and altering other gait parameters. Although promising, further investigation of a more clear relationship between manipulating multiple gait parameters and the effect on gait perception is still to be researched.

Future work on this study includes a larger scale public video rating system such as the one presented in this study, however with more videos covering a larger range of parameters such as further variation of knee location with a finer abnormality amplitude resolution. Although, we have moved one knee location to an asymmetric position, it would be interesting to examine if moving both knees equal distances provoke the same reactions in participants. These videos may also include studying the effects of altered limb thickness, texture, or limb form. We believe that the results of this and further investigations of what is considered normal human gait can help researchers, designers, and developers of gait modification devices, such as prosthetics or joint braces, create functionally better and more socially accepted devices. In this study we were only considering deviation of walking cadence and various parameter asymmetries, however further quantitative and qualitative investigation in the perception of the way the limbs move, that is, the limb angle trajectories (position, velocity, etc.).

### Conflict of interest statement

The authors declare that the research was conducted in the absence of any commercial or financial relationships that could be construed as a potential conflict of interest.

## Supplementary material

A Supplementary video of all gait patterns presented to participants is provided along with this manuscript. All the collected data and a Matlab® programming code used for interpretation is included as well. A snapshot image of the internet web page used to collect all perception data is included as well.
