# Supplementary material for: Perception of gait patterns that deviate from normal and symmetric biped locomotion
Source: Front Psychol. 2015 Feb 27;6:199. doi: 10.3389/fpsyg.2015.00199 (PMC4342886; doi:10.3389/fpsyg.2015.00199)
Supplement: Supplementary file 1 [file Presentation1.ZIP › Matlab programming code.DOCX]

%==============================================================================================================

% Ismet Handzic

% 08/2014

% REEDlab

% Department of Mechanical Engineering

% University of South Florida

%==============================================================================================================

%This code reads subject responses to gait videos collected over a website

%that are stored in a text file.

%The responses are stored in two columns in a text file "results.txt" in

%the following format:

%[Video#, Impairment, Uncanniness, Video#, Impairment, Uncanniness, Unused, Unique Subject Number]

%It uses a Chi^2 goodness of fit test to check if the data is normally

%distributed.

%It performs a Kruskal-Wallis statistical analysis for a 7-point Likert

%scale, while comparing which videos are statistically different with a

%post-hoc multiple comparison test.

%It finally compares two specified gait videos using a

%Mann-Whitney-Wilcoxon rank-sum test to determine if they stem from

%the same data distribution.

%==============================================================================================================

clc

clear all

close all

%==============================================================================================================

%Subjects video comparison number

%Only subjects above this threshold are considered

% Note: All subjects are loaded

ComparisonThresh = 1;

%Select Score Type

% Impairment ----> 0

% Uncanniness ----> 1

Scores = 0;

%Specify Videos to be included:

VideoNum = [1, 2, 3, 4, 5, 6, 7, 8, 9, 10, 11, 12, 13, 14, 15, 16, 17, 20, 21, 22, 23, 24, 25, 26, 27]; %All Videos

% VideoNum = [1, 27]; %Normal: Recorded (27) vs PDW (1)

% VideoNum = [1, 2, 3, 4, 5]; %Category 1 - Gait Speed

% VideoNum = [1, 6, 7, 8, 9, 10, 11]; %Category 2 - Knee Height

% VideoNum = [1, 12, 13, 14, 15, 16, 17]; %Category 3 - SL and SW Asym

% VideoNum = [1, 20, 21, 22]; %Category 4 - ROS

%VideoNum = [1, 23, 24, 25, 26]; %Category 5 - Knee Damping with Asym Mass

%Select the two specific videos to compare

% Note: Video numbers must be included in the above specified video number vector

VideoComp1 = 1;

VideoComp2 = 27;

%Load data from text file

DATA = csvread('Web_Rating_Data.csv');

%==============================================================================================================

fprintf('\n================ Videos and Subjects ==================')

VideoNum

%Delete duplicated and sort rows by subject

nonDuplicateindx = find(sum(abs(diff(DATA)),2) > 0);

DATA = sortrows(DATA(nonDuplicateindx,:), 8);

%Count subjects (all and unique)

SubjectsCount = 0;

SubjectsCountGood = 0;

Ratings = [];

DATA2 = [];

for i = 1: (length(DATA(:,8))-1)

if DATA(i,8) ~= DATA(i+1,8)

SubjectsCount = SubjectsCount + 1;

indexes = find( DATA(:,8) == DATA(i,8) );

%Store only data from subjects that completed enough comparisons

if length(DATA(indexes, 8)) > ComparisonThresh

SubjectsCountGood = SubjectsCountGood + 1;

%Number of Ratings

Ratings = [Ratings; SubjectsCountGood, length(indexes)*2];

%Renaming Subject Name/ID Number

NewSubjectNum = ones(length(indexes),1)*SubjectsCountGood;

DATA2 = [DATA2; DATA(indexes,1:7), NewSubjectNum];

end

end

end

%Fold Data over for rankings

% Format: [Video Number, Impairment, Uncanniness, Subject Number]

DATA_All = [DATA2(:,[1,2,3,8]); DATA2(:,[4,5,6,8])];

%Sort rows by subject

DATA_All = sortrows(DATA_All, 4);

fprintf('Total Subjects:\t\t\t\t\t%i\n', SubjectsCount)

fprintf('Valid Subjects (> %i trials):\t%i\n\n', ComparisonThresh, SubjectsCountGood)

%Loading only video numbers specified at the beginning

Intersection = ismember(DATA_All(:,1), VideoNum);

indexes = find(Intersection);

Data = DATA_All(indexes, :);

fprintf('Total Number of Video Ratings Considered: %i\n', length(Data))

fprintf('Median Ratings per Participant: %i\n', median(Ratings(:,2)))

%Seperating into variables

Video = Data(:,1);

Impairment = Data(:,2);

Uncanniness = Data(:,3);

Subject = Data(:,4);

%Selecting the type of scores specified at the beginning

if (Scores == 0)

ScoreCol = 2; %Impariment

else

ScoreCol = 3; %Uncanniness

end

Score = Data(:,ScoreCol);

%=============== Check if Data is Normally Distributed ====================

% Chi^2 goodness of fit

[Chi2, p1, stat_data] = chi2gof(Score);

fprintf('\n\n\n============ Chi^2 Goodness of Fit Test ===============\n')

if (Chi2 == 1)

fprintf('Data is *NOT* normally distributed.\n')

else

fprintf('Data is IS normally distributed.\n')

end

fprintf('dof = %g\n', stat_data.df)

fprintf('N = %g\n', SubjectsCountGood)

fprintf('X^2 = %g\n', stat_data.chi2stat)

fprintf('p = %g\n\n', p1)

fprintf('Format: X^2(%i, N=%i) = %g, p = %g\n\n', stat_data.df, SubjectsCountGood, stat_data.chi2stat, p1)

%============== Analysis of Varience (Kruskal-Vallis) =====================

fprintf('\n\nApplying Kruskal-Wallis One-way Analysis of Variances...\n')

[p2, table, stats] = kruskalwallis(Score, Video);

hold on

if (p2 < 0.01)

fprintf('Statistical difference found (p < 0.01).\n')

else

fprintf('Statistical difference *NOT* found (p > 0.01).\n')

end

fprintf('dof = %g\n', cell2mat(table(2,3)))

fprintf('N = %g\n', cell2mat(table(3,3)))

fprintf('H = %g\n', cell2mat(table(2,5)))

fprintf('p = %g\n', p2)

fprintf('Vector of Mean Ranks of Videos...')

stats.meanranks

fprintf('Format: H(%i, %i) = %g, p = %g\n\n', cell2mat(table(2,3)), cell2mat(table(3,3)), cell2mat(table(2,5)), p2)

%================== Find and Plot Averages ==============================

VideoAverage = [];

loop = 0;

for i = VideoNum

loop = loop + 1;

VideoAverage = [VideoAverage; mean( Data(find(Data(:, 1) == i), ScoreCol) )];

[i, i - VideoNum(2) + 2, VideoAverage(loop )];

plot(i - VideoNum(2) + 2, VideoAverage(loop), 'dr', 'markersize', 10, 'markeredgecolor', 'b', 'markerfacecolor', 'b');

plot(1, VideoAverage(1), 'dr', 'markersize', 10, 'markeredgecolor', 'b', 'markerfacecolor', 'b');

end

plot(length(VideoNum), VideoAverage(end), 'dr', 'markersize', 10, 'markeredgecolor', 'b', 'markerfacecolor', 'b');

%=========================== Format Plot ==================================

%Paint All BOX Lines

all_box_line_h = findobj('type','line','Marker','none',...

'-and','-not','LineStyle',':', '-and','color', 'b');

set(all_box_line_h, 'Linewidth', 3, 'color', [0/255, 200/255, 0/255]); %GREEN

%Paint All MEDIAN Lines

all_median_line_h = findobj('type','line','Marker','none',...

'-and','-not','LineStyle',':', '-and','color', 'r');

set(all_median_line_h, 'Linewidth', 4);

%Paint All RANGE Lines

all_range1_line_h = findobj('type','line','Marker','none',...

'-and','-not','LineStyle','-', '-and','color', 'k');

set(all_range1_line_h, 'Linewidth', 3);

all_range2_line_h = findobj('type','line','Marker','none',...

'-and','LineStyle','-', '-and','color', 'k');

set(all_range2_line_h, 'Linewidth', 3);

%Paint All OUTLIER Markers

all_marker_h = findobj('type','line','Marker','+');

for i = 1:length(all_marker_h)

set(all_marker_h(i), 'Markersize', 5, 'color', 'k', 'MarkerEdgeColor', 'r', 'MarkerFaceColor', 'r', 'Marker', 'o');

end

%Make lines Neutral Line at 4

plot([0 length(VideoNum)+1], [4 4], '-.k', 'linewidth', 2.5, 'color', [0.3, 0.3, 0.3])

TopTitle = 'Kruskal-Wallis Statistical Analysis';

BottomTitle = sprintf('%i Participants', SubjectsCountGood);

title({TopTitle; BottomTitle}, 'FontSize' ,18, 'FontName','Times New Roman');

xlabel('Video Number','FontSize',14, 'FontName','Times New Roman');

ylabel('Likert Scale','FontSize',14, 'FontName','Times New Roman');

set(gca, 'YGrid', 'On')

grid on

set(gca, 'YLim', [1, 7])

%=========== Multiple Comparison Post-hoc Analysis ======================

fprintf('\nApplying Multiple Comparison Post-Hoc Analysis...\n')

hold off

figure;

%First combines both data vectors into one

%It then compares the mean (average) ranks for each data set

[c, m, h, nms] = multcompare(stats, 'estimate', 'kruskalwallis');

hold on

grid on

%================ Compare Two Specified Gait Videos =======================

Video1 = Data( find(Data(:,1) == VideoComp1), ScoreCol);

Video2 = Data( find(Data(:,1) == VideoComp2), ScoreCol);

%Mann–Whitney U-test / Mann–Whitney–Wilcoxon test / Wilcoxon rank-sum test / the Wilcoxon two-sample test

% Mann-Whtney-Wilcoxon Rank-Sum Test first combines both data vectors into one

% It then compares the mean (average) ranks for each data set

[p2, h2, stats2] = ranksum(Video1, Video2, 'method', 'approximate');

n1 = length(Video1);

n2 = length(Video2);

fprintf('\n\n========= Mann-Whitney-Wilcoxon rank-sum test =========\n')

if (h2 == 1) %rejects null hypothesis of equal mean ranks at 5% sig. level (statistically different)

fprintf('Statistical difference detected between Video %i and Video %i.\n', VideoComp1, VideoComp2)

else %does not rejects null hypothesis of equal mean ranks at 5% sig. level (statistically same)

fprintf('*NO* statistical difference detected between Video %i and Video %i.\n', VideoComp1, VideoComp2)

end

fprintf('n1 = %g\n', n1)

fprintf('n2 = %g\n', n2)

fprintf('p = %g\n', p2)

fprintf('Z = %g\n', stats2.zval)

fprintf('Rank Sum (r) = %g\n\n', stats2.ranksum)

%==============================================================================================================

%==============================================================================================================

%==============================================================================================================
