# Supplementary figures and images for: Perception of gait patterns that deviate from normal and symmetric biped locomotion
Source: Front Psychol. 2015 Feb 27;6:199. doi: 10.3389/fpsyg.2015.00199 (PMC4342886; doi:10.3389/fpsyg.2015.00199)

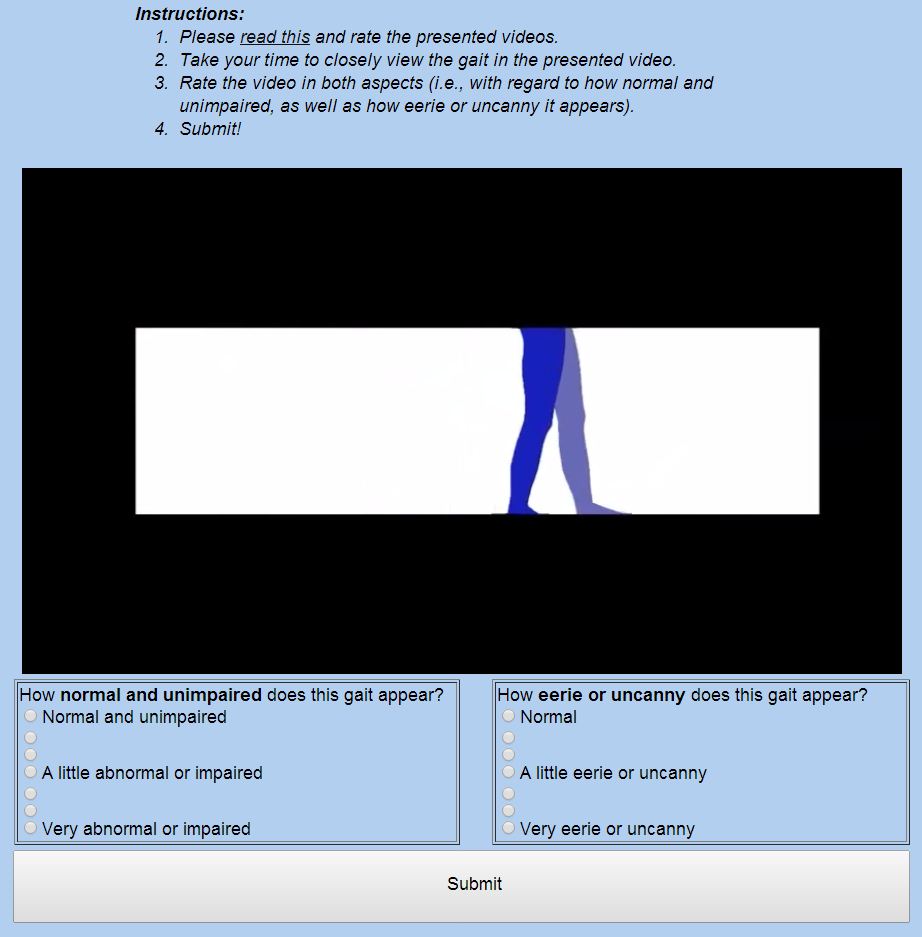

Supplement: Supplementary file 1 [file Presentation1.ZIP › Supplementary Image.JPEG]
